# Pharmacological inhibition of REV-ERBα ameliorates motor deficits in the 6-OHDA-lesioned mouse model of Parkinson’s disease

**DOI:** 10.1080/19768354.2026.2690728

**Published:** 2026-10-15

**Authors:** Jeongah Kim, Mijeong Choe, Sangwon Jang, Inah Park, Sungjoon Oh, Eun-Kyoung Kim, Youngshik Choe, Woong Sun, Kyungjin Kim

**Affiliations:** a Department of Pharmacology, College of Medicine, Konkuk University, Chungju, Korea; b Department of Brain Sciences, Daegu Gyeongbuk Institute of Science and Technology (DGIST), Daegu, Korea; c Developmental Disorders & Rare Diseases Group, Korea Brain Research Institute (KBRI), Daegu, Korea; d Department of Anatomy, College of Medicine, Korea University, Seoul, Korea

**Keywords:** Parkinson's disease, REV-ERBα, nigrostriatal dopamine system

## Abstract

Parkinson’s disease (PD) is characterized by nigrostriatal dopaminergic (DAergic) degeneration and motor dysfunction. Many PD patients also show circadian rhythm disturbances. Increasing evidence links circadian clock dysfunction to PD pathology. REV-ERBα is a circadian nuclear receptor that regulates DAergic genes under physiological conditions. However, its role in nigrostriatal dopamine (DA) homeostasis and motor function in PD remains unclear. In this study, we found elevated Rev-erbα mRNA expression in tyrosine hydroxylase (TH)-positive neurons of the substantia nigra (SN) in a 6-hydroxydopamine (6-OHDA) mouse model of PD. To test its functional role, we assessed the acute effects of local SN microinjection of the REV-ERBα antagonist SR8278. We found that SR8278 partially recovered motor performance and improved fine motor function. These effects occurred regardless of circadian timing. SR8278 administration did not restore TH expression or tissue DA levels in the striatum and SN. However, in vivo microdialysis showed enhanced extracellular DA turnover in the striatum. Specifically, while extracellular DA metabolites level increased, basal extracellular DA levels showed no change. Together, these findings identify REV-ERBα as a potential therapeutic target that modulates extracellular nigrostriatal DA dynamics and improves motor function in PD.

## Introduction

Parkinson’s disease (PD) is one of the most common neurodegenerative disorders. It is characterized by the selective loss of dopaminergic (DAergic) neurons in the substantia nigra (SN), which leads to striatal dopamine (DA) depletion. Although PD has been studied for decades, its underlying pathogenic mechanisms remain incompletely understood, and no curative treatment is available. Clinically, PD manifests as motor symptoms such as bradykinesia, rigidity, and tremor, along with a wide range of non-motor symptoms. These include sleep and mood disturbance, which have been linked to circadian dysregulation (Breen et al. 2014, Videnovic et al. 2014, Leng et al. 2019).

The circadian clock coordinates daily physiological and behavioral processes through endogenous molecular oscillators (Vitaterna et al. 2001, Ruan et al. 2021). It operates through interlocked transcriptional-translational feedback loops composed of clock genes. REV-ERBα (also known as NR1D1) is a core component of the mammalian molecular clock and functions as a transcriptional repressor. REV-ERBα regulates diverse physiological and pathological processes, including metabolism (Cho et al. 2012, Solt et al. 2012, Adlanmerini and Lazar 2023), mitochondrial function (Woldt et al. 2013), and neuroinflammatory responses (Kim et al. 2018, Griffin et al. 2019). REV-ERBα also plays an important role in modulating DA synthesis and signaling in the brain. Rev-erbα knockout (KO) mice show increased DAergic gene expression and DA turnover in the hippocampus(Jager et al. 2014). In the ventral tegmental area (VTA), REV-ERBα regulates the circadian expression of tyrosine hydroxylase (TH), the rate-limiting enzyme in DA biosynthesis. This regulations occurs through an antagonistic interaction with Nurr1, a nuclear receptor that is essential for DAergic neuronal function (Chung et al. 2014).

Circadian dysfunction is increasingly recognized as a potential risk factor for PD pathogenesis (Videnovic, et al. 2014, Shkodina et al. 2022). REV-ERBα has attracted attention because of its involvement in multiple pathological conditions (Wang et al. 2020). In 1-methyl-4-phenyl-1,2,3,6-tetrahydropyridine (MPTP)-induced models of DAergic neurodegeneration, chronic treatment with a REV-ERBα agonist produced anti-inflammatory and neuroprotective effects through acting on microglia, leading to behavioral recovery (Kou et al. 2022, Gupta et al. 2024). Conversely, pharmacological inhibition of REV-ERBα reduced MPTP-induced DAergic neuronal ferroptosis, a form of cell-death implicated in PD, and protected against neuronal damage (Wang et al. 2024). We previously reported that microinjection of the REV-ERBα antagonist, SR8278 restored circadian-dependent mood-related behaviors in a 6-hydroxydopamine (6-OHDA)-lesioned PD model (Kim et al. 2022). SR8278 reduced depressive- and anxiety-like behaviors at specific time points in the 6-OHDA-lesioned mice. These effects were associated with restoration of REV-ERBα–NURR1 antagonistic regulation and recovery of rhythmic TH expression in the VTA.

Despite these findings suggesting REV-ERBα as a potential therapeutic target for PD, how REV-ERBα contributes to the regulation of nigrostriatal DA homeostasis under parkinsonian conditions remains unclear. In this study, we investigated how acute pharmacological modulation of REV-ERBα affects nigrostriatal DA homeostasis in a 6-OHDA mouse model of PD. We examined the effects of modulation of REV-ERBα on DA synthesis, tissue DA content, and extracellular DA turnover, and assessed how these changes related to motor behaviors in these mice. 

## Material and methods

### Unilateral 6-OHDA model

Male C57BL/6J wild-type mice were maintained under controlled conditions with a 12 h light/dark cycle (lights on at 10:00 am) at 22–23 °C, with free access to food and water. All animal procedures were approved by the Institutional Animal Care and Use Committee of DGIST (DGIST-IACUC-20102305–000). A unilateral striatal 6-OHDA lesion model of PD was established as previously described (Kim, et al. 2022). Mice were anesthetized with pentobarbital sodium (50 mg/kg, i.p.) and placed in a stereotaxic apparatus (Stoelting). 6-OHDA (15 µg in 1.0 µL saline containing 0.02% ascorbic acid) or vehicle was injected into the left dorsal striatum (AP +0.6 mm, ML −2.0 mm, DV −3.5 mm relative to bregma) at a flow rate of 0.5 µL/min using a 26-gauge microsyringe.

### MPTP model

Mice were subjected to the MPTP-induced PD model as previously described (Jackson-Lewis and Przedborski 2007), with minor modifications. Mice received four intraperitoneal injections of MPTP (14 mg/kg) administered at 2-h intervals.

### Microinjection of SR8278

SR8278 was microinjected into the ventral midbrain toward the SN 3 h before each behavioral test under dim red-light conditions. The timing of drug administration relative to behavioral testing and general animal handling procedures were adapted from a previously established protocol (Kim, et al. 2022). SR8278 (Tocris Bioscience) was dissolved in ethanol at a final concentration of 50 µg/µL. SR8278 (20 µg/mouse) or an equivalent volume of vehicle (ethanol) was unilaterally infused using a 33-gauge injector cannula (model C315I; Plastics One) connected to a 10-µL Hamilton syringe at a constant rate of 0.1 µL/min. For microinfusion procedure, mice were anesthetized with sodium pentobarbital (50 mg/kg, i.p.) and secured in a stereotaxic frame (Stoelting). A 26-gauge stainless steel guide cannula (model C315G; Plastics One) was implanted into the ventral midbrain toward the SN (AP −3.2 mm, ML −1.0 mm, DV −4.5 mm relative to bregma). A 32-gauge dummy cannula was inserted to maintain patency. The cannula assembly was anchored to the skull using jewelry screws and fixed with dental resin cement.

### Behavioral tests

Five weeks after injection, mice were transferred to constant darkness for 3–4 days to allow acclimatization and to minimize the influence of light, starting from the onset of the dark phase. All behavioral tests were subsequently performed under dim red light.

### Cylinder test

Forelimb-use asymmetry was assessed using the cylinder test, as previously described (Kim, et al. 2022). Mice were individually placed in a transparent glass cylinder (11 cm diameter, 30 cm height) for 5 min, and forepaw contacts during rearing and landing were recorded. Forelimb preference was calculated as [R/(L + R)] × 100, where R and L indicate the number of right and left forepaw contacts, respectively.

### Rotarod test

Motor coordination was evaluated using an accelerating rotarod apparatus (B.S. Technolab Inc.) following an established protocol (Kim, et al. 2022). Mice were trained on the rotarod at a constant speed of 4 rpm for 5 min and allowed to rest for at least 60 min. Mice then underwent three test trials separated by at least 30 min. During each trial, the rotation speed increased from 4 to 40 rpm over 5 min, and the latency to fall was recorded. The mean latency from three trials was used for statistical analysis.

### Pole test

Motor coordination and bradykinesia were assessed using the pole test in MPTP-treated mice. Mice were placed head-upward on the top of a vertically positioned rough-surfaced pole, and the total time required to descend to the base of the pole was recorded. Trials in which mice slipped or jumped from the pole were excluded and repeated after a brief rest period. Each mouse completed three trials with an inter-trial interval of at least 5 min. The mean value was used for statistical analysis. A maximum cut-off time of 60 s was applied for each trial.

### Western blot

Mice were euthanized and SN region were rapidly dissected, snap-frozen on dry ice and stored at −70 °C until analysis. Western blotting was performed as previously described (Kim, et al. 2022). Briefly, protein lysates were separated by SDS–polyacrylamide gel electrophoresis and transferred onto PVDF membranes (Millipore) using a Bio-Rad Trans-Blot system with Towbin transfer buffer (25 mM Tris [pH 8.3], 192 mM glycine, 20% methanol). Membranes were blocked in Tris-buffered saline (TBS; 10 mM Tris [pH 7.6], 150 mM NaCl, 2 mM MgCl₂) supplemented with 0.3% Tween-20 and 5% bovine serum albumin (BSA) and incubated with primary antibodies against tyrosine hydroxylase (TH; T2928, Sigma-Aldrich) and β-actin (sc-47778, Santa Cruz Biotechnology) at room temperature for 1 h. After washing four times with TBS containing 0.3% Tween-20, membranes were incubated with horseradish peroxidase–conjugated secondary antibodies (Jackson ImmunoResearch). Immunoreactive signals were detected using enhanced chemiluminescence reagents (Thermo Fisher Scientific) and visualized using X-ray film or using a ChemiDoc XRS imaging system (Bio-Rad).

### Immunohistochemistry

Mice were deeply anesthetized with pentobarbital sodium (50 mg/kg, i.p.) and transcardially perfused with phosphate-buffered saline (PBS, pH 7.6) followed by 4% paraformaldehyde. Brains were removed, post-fixed overnight and cryoprotected in 30% sucrose at 4 °C for 48 h. Coronal brain sections (20 µm) were prepared using a cryostat (Leica). Sections were subjected to antigen retrieval in sodium citrate buffer at 100 °C for 10 min, followed by several washes in PBS containing 0.1% Triton X-100. Sections were then blocked with PBS containing 0.3% Triton X-100 and 10% normal goat serum for 60 min at room temperature. After washing, sections were incubated overnight at 4 °C with an anti-tyrosine hydroxylase (TH) antibody (Abcam, ab112). Following additional washes, sections were incubated with a biotinylated secondary antibody for 1 h, and immunoreactivity was visualized using an avidin–biotin–peroxidase complex (Vectastain ABC kit; Vector Laboratories) and 3,3′-diaminobenzidine as the chromogen. TH-stained sections were examined using an Eclipse 90i microscope (Nikon).

### RNA isolation and real-time qPCR

Tissues from the ipsilateral side of the lesion were collected for RNA analysis. Total RNA was extracted using TRIzol reagent (Invitrogen) according to the manufacturer’s protocol. For RT–PCR, 1 μg of each RNA sample was reverse-transcribed using the PrimeScript RT reagent (Takara Bio). Aliquots of the cDNA were then subjected to real-time qPCR in the presence of SYBR Green I (Enzynomics). Gene expression levels were normalized to the gene encoding TATA-box binding protein (Tbp). VMAT2-F: 5’-TTCTTCGAAGTCCACCTGCT-3’, VMAT-R: 5’-ACATTGGGCAACGTTAGAGG-3’, DAT-F: 5’-CCTGGTTCTACGGTGTTCAG-3’, DAT-R: 5’-GCTGACCACGACCACATACA-3, DRD2-F: 5’-TATGGCTTGAAGAGCCGTGC-3’ DRD2-R: 5’-CCCTTCGGACCCATTGAAGG-3’, TBP-F: 5’-GGGAGAATCATGGACCAGAA-3’, TBP-R: 5’-CCGTAAGGCATCATTGGACT-3’.

### Fluorescence in situ hybridization (FISH)

At the indicated time points, mouse brains were rapidly dissected and snap-frozen in isopentane precooled on dry ice. Coronal cryosections (14 µm) containing the ipsilateral SN were prepared and mounted on Superfrost Plus slides. Sections were fixed in 4% paraformaldehyde for 10 min, dehydrated through graded ethanol, air-dried, and pretreated with protease for 10 min at room temperature. RNAscope probes against Th (Mm-Th-C3, Cat. No. 317621-C3), Nurr1 (Mm-Nr4a2-C1, Cat. No. 423351), and Rev-erbα (Mm-Nr1d1-C2, custom probe targeting nucleotides 301–1404 of NM_145434.4; Advanced Cell Diagnostics) were used. Probe hybridization, signal amplification, and detection were performed as previously described (Kim, et al. 2022) using a HybEZ hybridization oven at 40 °C. Images were acquired using a LSM 800 confocal microscope (Zeiss) with a Plan-Apochromat 20× objective (NA = 0.8). Quantification was performed within the SNpc in coronal sections corresponding to bregma −3.16 mm (AP), 1.2 mm (ML) and 4.3 mm (DV), based on the Allen Mouse Brain Atlas. Two coronal sections encompassing the SNpc were analyzed per animal. Images were analyzed using ImageJ software (NIH). The ROI was defined based on TH-positive signals and DAPI nuclear counterstaining. ROI selection and subsequent quantification were performed in a blinded manner with respect to experimental conditions. All quantitative analyses were performed using low-magnification images (159.73 μm × 159.73 μm field of view). TH-positive cell counts were quantified from low-magnification images. Rev-erbα mRNA and Nurr1 mRNA puncta were independently quantified within TH-positive ROIs using the Analyze Particles function in ImageJ. Transcript numbers for each target were normalized to the number of TH-positive neurons and expressed as the average number of transcripts per TH-positive neuron.

### High-performance liquid chromatography-electrochemical detection (HPLC-ECD)

DA and its metabolites were measured in the striatum and SN. Mouse brains were sectioned into 2-mm coronal slices using a brain matrix. The striatum was isolated by micropunching (2 mm diameter) and the SN region was dissected manually using a blade. Tissues were stored at −80 °C until further processing. Brain tissues were washed with prechilled phosphate-buffered saline and homogenized in 0.3 N perchloric acid using a sonicator with pulses at 12 volts on ice. The homogenates were centrifuged at 20,000 × g for 10 min at 4 °C, and the resulting supernatants were collected for HPLC-ECD analysis.

Quantitative analysis of monoamines and their metabolites was performed using a Dionex Ultimate 3000 HPLC system (Thermo Fisher Scientific) equipped with a pump and autosampler and coupled to an electrochemical detector. Chromatographic separation was achieved using a BDS Hypersil C18 column (150 × 3 mm, 3 μm; Thermo Fisher Scientific) with a corresponding guard column. A commercially available TEST mobile phase (Thermo Fisher Scientific) was used according to the manufacturer’s instructions. The flow rate was set at 0.5 mL/min, the column oven temperature was maintained at 30°C, and 10 μL of sample was injected for each analysis. The electrochemical detector was performed using a 6011RS Ultra analytical cell (Thermo Scientific Dionex) at 250 and 400 mV. Quantitative values obtained at the two potentials were averaged for quantitative analysis. Owing to extremely small tissue amounts, monoamine levels were expressed as absolute amounts (ng/sample) without normalization to tissue weight.

### In vivo microdialysis

In vivo microdialysis was performed in freely moving mice to measure extracellular DA and its metabolites in the striatum. A CMA7 guide cannula was stereotaxically implanted into the left striatum (AP +0.6 mm, ML −2.0 mm, DV −3.5 mm relative to bregma). A CMA7 microdialysis probe (6 kDa cutoff, 2 mm membrane length) was inserted through the guide cannula for sampling. For SR8278 administration, an additional cannula was implanted at a 10° angle (AP: −3.82 mm; ML: −1.0 mm; DV: −4.55 mm). Artificial cerebrospinal fluid (aCSF; Tocris; final ion concentrations in mM: Na^+^ 150, K^+^ 3.0, Ca²^+^ 1.4, Mg²^+^ 0.8, P 1.0, Cl^−^ 155) was perfused through the microdialysis probe at a flow rate of 1 μL/min using FEP tubing. For allow free movement during sampling, a low-torque dual-channel quartz-lined microdialysis swivel (model 375/D/22QM, INSTECH) and a counter-balance arm (9 cm; model SMCLA, INSTECH) were used. Dialysates were collected at 1-h intervals (60 μl per fraction) into microcentrifuge tubes preloaded with 6.6 μL of 1 N perchloric acid (Sigma-Aldrich), and kept on ice during sampling, resulting in a final perchloric acid concentration of 0.1 N. Samples were stored at −80 °C until analysis.

Monoamines and their metabolites were quantified using an Agilent 1290 Ultra-Performance Liquid Chromatography system coupled to an Agilent 6490 Triple Quadrupole mass spectrometer operated in electrospray ionization (ESI–MS/MS) mode. The mass spectrometer was operated in multiple reaction monitoring (MRM) mode with individually optimized precursor–product ion transitions for each analyte. Chromatographic separation was performed on a Phenomenex Synergi Hydro-RP column (150 × 2.0 mm, 4 μm, 80 Å) maintained at 45 °C. The mobile phase consisted of 10 mM ammonium acetate containing 0.1% formic acid in water (solvent A) and acetonitrile (solvent B). The gradient was programed as follows: 0–1.5 min, 0% B; 1.5–9 min, linear increase to 90% B; 9–9.1 min, return to initial conditions; and 9.1–12 min, re-equilibration. The flow rate was maintained at 0.3 mL/min, and the injection volume was 10 μL. DA-d4 was used as an internal standard for quantification.

### Statistics

All data were reported as mean ± SEM. Statistical analyses were performed using GraphPad Prism (version 8.0; GraphPad Software). Group differences were evaluated using a two-way analysis of variance (ANOVA) followed by a post-hoc comparison using Tukey’s test or a two-way repeated measured ANOVA followed by a post-hoc comparison using a Fisher's LSD. A value of *p* < 0.05 was considered statistically significant.

## Results

### Rhythmic Rev-erbα mRNA expression is disrupted in DAergic neurons of the substantia nigra pars compacta (SNpc) after 6-OHDA lesion

To examine Rev-erbα expression in nigrostriatal DAergic neurons under PD conditions, we used a unilateral intrastriatal 6-OHDA mouse model. Five weeks after lesioning, mice showed a major loss of TH-positive fibers in the striatum and TH-positive neurons in the SNpc, confirming successful induction of the lesion (Figure 1A). To assess DAergic neuron–specific expression of Rev-erbα in the SNpc, we performed fluorescence in situ hybridization (FISH) for Rev-erbα together with Nurr1, a key transcription factor in DAergic neurons (Figure 1B and C). To evaluate circadian variation, we analyzed gene expression at two circadian time (CT) points: subjective dawn (CT00) and subjective dusk (CT12). Quantitative of FISH signals confirmed a significant reduction in TH-positive neurons in the SNpc after 6-OHDA lesion, regardless of circadian time (Figure 1D). In contrast, the VTA was substantially less affected and showed only a mild reduction in TH-positive cell counts at CT12 (Supplementary Fig. S1). This result is consistent with the relatively resistance of the VTA to 6-OHDA-induced neurodegeneration. In sham mice, Rev-erbα mRNA showed a clear diurnal rhythm in TH-positive neurons of the SNpc, with peak expression at CT12 (Figure 1E). In 6-OHDA-lesioned mice, this rhythm was lost, and Rev-erbα expression remained elevated at CT00. In contrast, Nurr1 mRNA showed no rhythmic pattern in sham mice but became rhythmic after 6-OHDA lesion (Figure 1F). These results indicate that the normal circadian relationship between Rev-erbα and Nurr1 is disrupted in nigrostriatal DAergic neurons after 6-OHDA-lesion.

**Figure 1.**
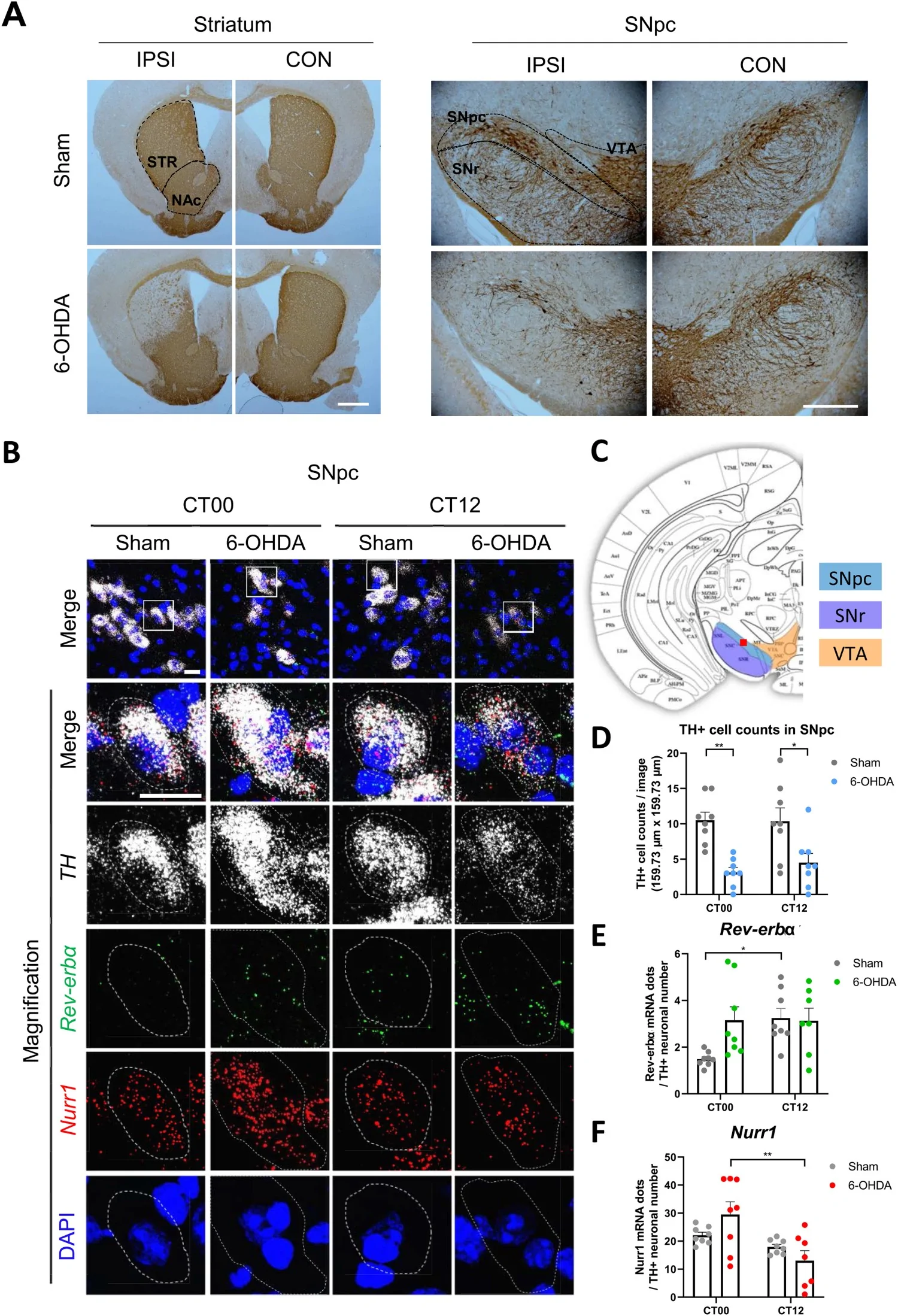
**Rev-erbα mRNA is elevated in TH-positive DAergic neurons of the SNpc after 6-OHDA-lesion and loses its circadian rhythm.** (A) Representative TH immunostaining of the striatum and SNpc in sham- and 6-OHDA-lesioned mice five weeks after lesioning. Scale bar, 200 μm. STR, striatum; NAc, nucleus accumbens; SNpc, substantia nigra pars compacta; SNr, substantia nigra pars reticulata; VTA, ventral tegmental area. (B) Representative fluorescence in situ hybridization (FISH) images showing Rev-erbα and Nurr1 mRNA puncta in TH-positive neurons of the SNpc at CT00 and CT12. White boxes indicate regions shown at higher magnification. Scale bar, 20 μm. (C) Anatomical schematic indicating the imaging and quantification region within the SNpc. (D) Quantification of TH-positive neurons in the SNpc (N = 8). (E and F) Quantification of Rev-erbα and Nurr1 mRNA puncta normalized to TH-positive neuron number (N = 7-8). Data are presented as mean ± SEM with individual values shown. Statistical significance was determined by two-way ANOVA followed by Tukey’s post hoc analysis. *p < 0.05, **p < 0.01.

### SR8278 administration rescues fine motor behavior but not motor coordination in 6-OHDA-lesioned mice

Because Rev-erbα mRNA expression was significantly elevated in TH-positive neurons of the SN after 6-OHDA-lesion (Figure 1E), we examined whether pharmacological inhibition of REV-ERBα affects motor behavior. SR8278 was locally microinjected into the SN 3 h before behavioral testing (Figure 2A). Behavioral tests were performed at subjective dawn (CT22-01) and subjective dusk (CT10-13) to assess potential time-of-day effects. In the cylinder test, 6-OHDA-lesioned mice showed a significantly reduced use of contralateral forelimb compared with sham controls, consistent with unilateral DAergic dysfunction (Figure 2B). Acute local administration of SR8278, into the SN significantly restored forelimb use asymmetry shown in 6-OHDA lesioned mice. This effect was observed at both CT22-01 and CT10-13, indicating that the SR8278 action was independent of circadian time. In contrast, in the rotarod test, which assesses motor coordination and balance, latency to fall did not differ between sham and 6-OHDA-lesioned mice at either time points (Figure 2C). SR8278 treatment also had no effect on rotarod performance. These findings indicate that REV-ERBα inhibition improves fine motor behavior but does not affect overall motor coordination.

**Figure 2.**
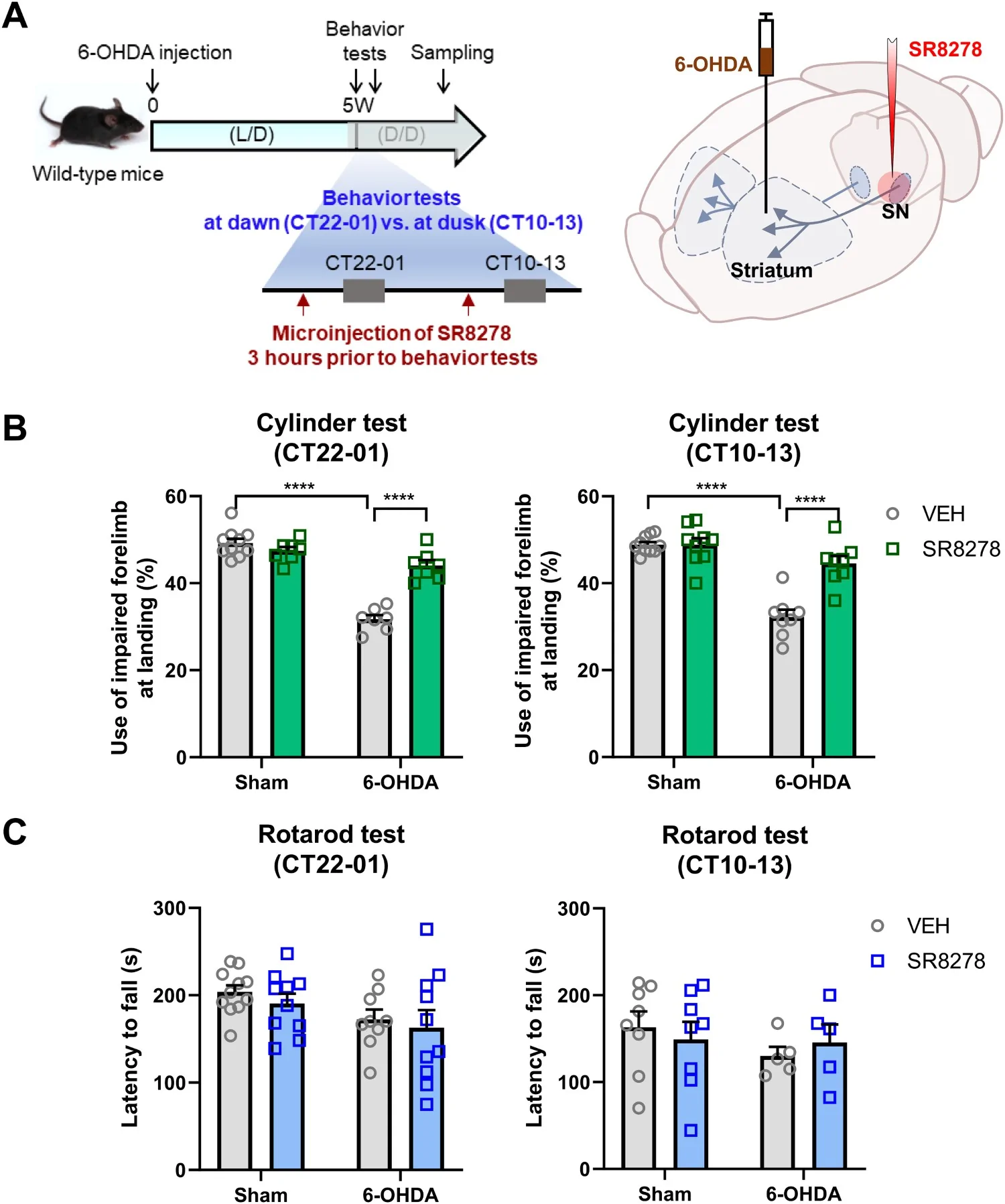
**REV-ERBα antagonism improves fine motor performance in striatal 6-OHDA lesion independent of circadian time.** (A) Experimental design. Mice received unilateral intrastriatal injection of 6-OHDA. After 5 weeks of 6-OHDA lesioning, behavioral testing was performed at circadian time (CT) 22–01 or CT10-13. SR8278 was microinjected into the SN 3 h before behavioral testing. (B) Cylinder test analysis of contralateral forelimb use in sham- and 6-OHDA-lesioned mice treated with VEH or SR8278 (N = 7–10 at CT22-01; N = 8–10 at CT10–13) (C) Rotarod performance measured as latency to fall in sham- and 6-OHDA-lesioned mice treated with VEH or SR8278 microinjection (N = 9–11 at CT22-01; N = 5–8 at CT10-13). Data are represented as mean ± SEM. Individual dots represent biological replicate. Statistical significance was determined by two-way ANOVA followed by Tukey’s post hoc analysis. ****p < 0.0001.

To determine whether this effect extends to another PD model, we tested SR8278 in the acute MPTP mouse model using the pole test (Supplementary Fig. S2A). MPTP-treated mice showed impaired motor performance, as indicated by longer descent latency (Supplementary Fig. S2B). SR8278 treatment reduced this latency and improved performance in MPTP-treated mice. Together, these results suggest that local SR8278 administration into the SN improves fine motor function across two mouse models of PD.

### Acute SR8278 injection does not restore TH or other DAergic markers in the SN

To determine whether SR8278-induced motor improvement was associated with changes in DA synthesis-related pathways, we examined TH expression as TH is the rate-limiting enzyme for DA biosynthesis. Western blot analysis showed reduced TH expression in the ipsilateral SN of 6-OHDA lesioned mice compared with sham controls (Figure 3A, B). No change was observed in the contralateral SN (Supplementary Fig. S3). SR8278 treatment did not restore TH protein levels in the lesioned SN (Figure 3A, B). To determine whether other components of the DAergic phenotype were affected, we next examined the expression of other DAergic markers, such as vesicular monoamine transporter 2 (VAMT2), dopamine transporter (DAT), and dopamine receptor D2 (DRD2) in the SN. The expression of all three markers was reduced after 6-OHDA lesion (Figure 3C-E). SR8278 treatment did not alter their expression. These findings indicate that motor improvement produced by SR8278 is unlikely not mediated by transcriptional restoration of TH or other DAergic markers in the SN.

**Figure 3.**
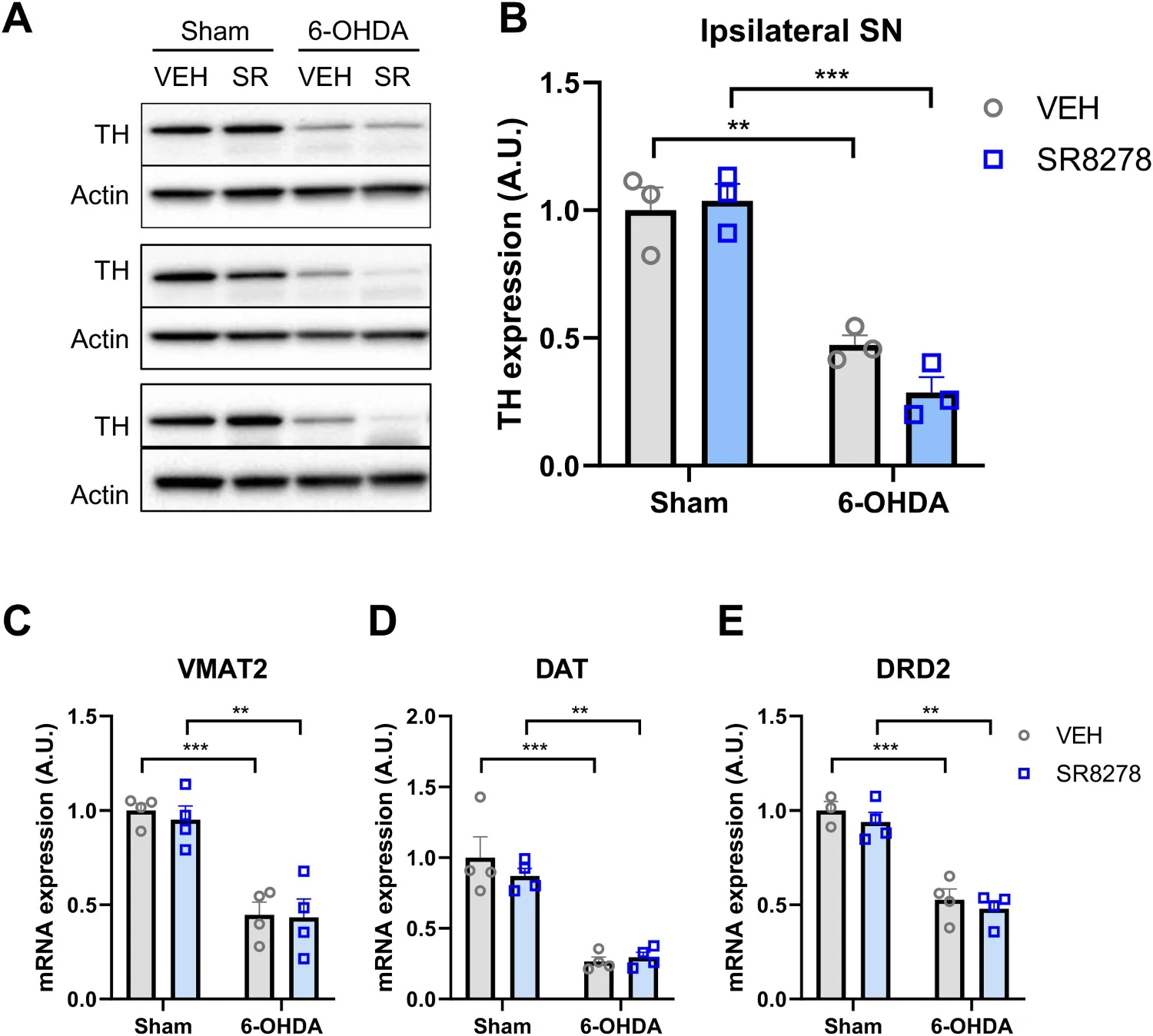
**SR8278 treatment does not restore the expression of TH or other DAergic markers in the SN of 6-OHDA-lesioned mice.** (A) Representative western blot images of TH in the SN from sham- and 6-OHDA-lesioned mice treated with VEH or SR8278. β-actin was used as a loading control. Three independent sets are shown. (B) Densitometric quantification of TH protein levels normalized to β-actin. (C-E) Quantitative RT-PCR analysis of VMAT2, DAT, and DRD2 mRNA expression in the SN from sham- and 6-OHDA-lesioned mice treated with VEH or SR8278. For all experiments shown in this figure, SR8278 was administered 3 h prior to tissue collection, and SN tissues were collected at CT22–01. Data are presented as mean ± SEM, with individual values shown (n = 3 for B; n = 3–4 for C–E). Statistical significance was determined by two-way ANOVA followed by Tukey’s post hoc analysis. **p < 0.01, ***p < 0.001.

### Acute SR8278 treatment does not alter DA content or DA turnover in the striatum and SN

To investigate whether SR8278 affects DA metabolism, we measured tissue levels of DA and its metabolites using HPLC-ECD. Because 6-OHDA was injected into the left striatum and SR8278 into the left SN, we analyzed both regions on the lesioned side. DA content was reduced in the striatum of 6-OHDA-lesioned mice compared with sham controls, while the SN exhibited a smaller and less pronounced trend toward a decrease (Figure 4A and E). SR8278 treatment did not alter DA levels in either region. In the striatum, DOPAC and HVA levels did not differ among groups (Figure 4B and C), indicating that neither the 6-OHDA lesion nor SR8278 treatment affected these metabolites. In the SN, DOPAC levels were unchanged (Figure 4F), whereas HVA levels were reduced after 6-OHDA-lesion (Figure 4G). SR8278 treatment did not affect either metabolite (Figure 4B, C, F, G). The DA turnover rate, defined as (DOPAC + HVA)/DA, showed a modest increase in the striatum after 6-OHDA-lesion (Figure 4D). No group differences were observed in the SN (Figure 4H). SR8278 administration did not affect DA turnover in either region (Figure 4D and H). Analysis of the contralateral striatum and SN showed no differences among groups (Supplementary Fig. S4), confirming 6-OHDA lesion-induced changes were restricted to the ipsilateral side. Together these data indicate that acute SR8278 treatment does not alter basal tissue DA content, DA metabolite levels, or tissue DA turnover in the striatum or SN. We therefore next examined whether SR8278 modulates extracellular DA dynamics using in vivo microdialysis.

**Figure 4.**
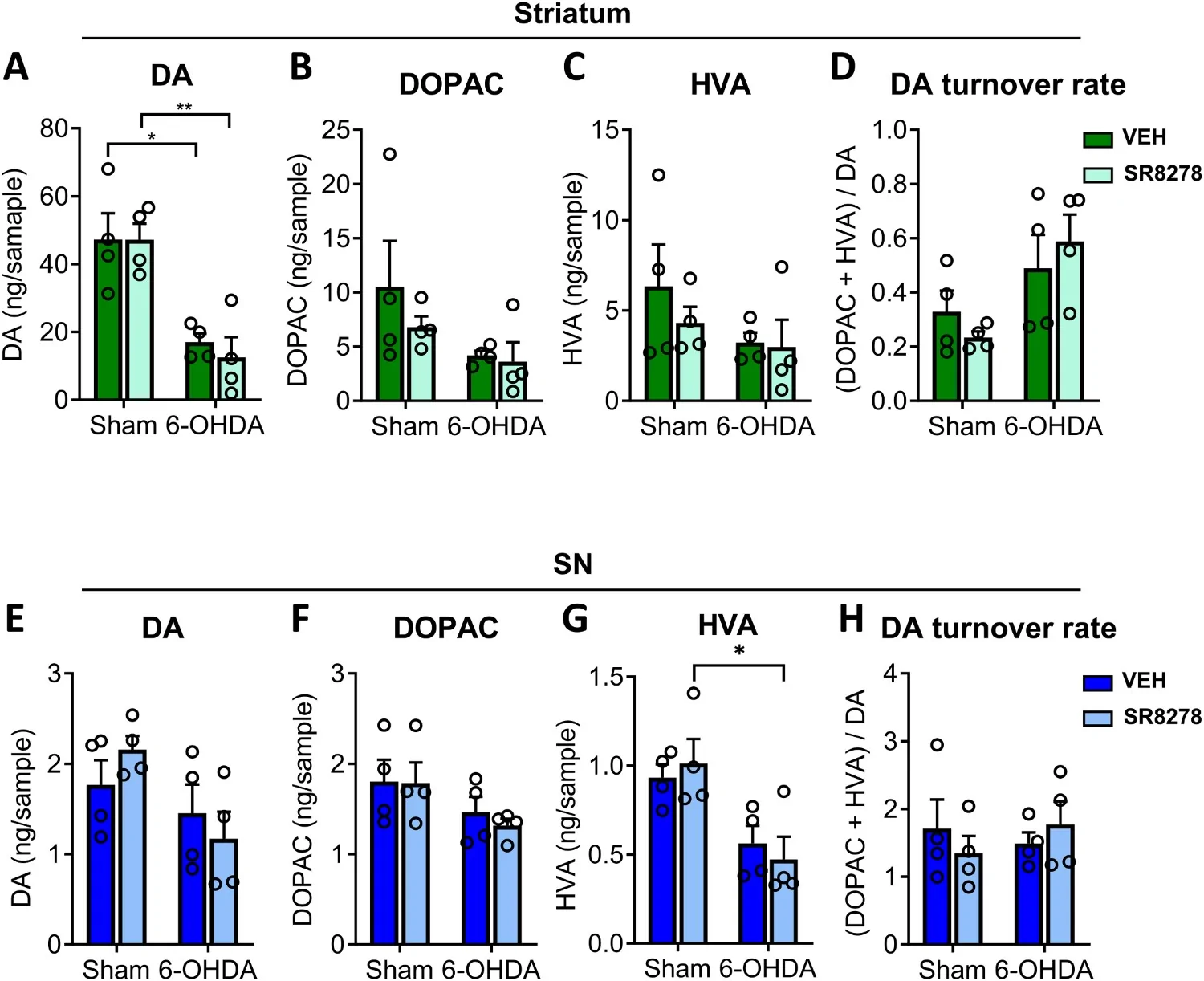
**SR8278 does not restore tissue DA content or DA metabolites levels in the striatum and SN of 6-OHDA-lesioned mice.** (A-D) HPLC-ECD analysis of tissue DA, DOPAC, HVA, and DA turnover ratio ((DOPAC + HVA)/DA) in the ipsilateral striatum from sham- and 6-OHDA-lesioned mice treated with VEH or SR8278. (E-H) HPLC-ECD analysis of tissue DA, DOPAC, HVA, and DA turnover ratio in the SN from sham- and 6-OHDA-lesioned mice treated with VEH or SR8278. SR8278 was administered 3 h prior to tissue collection, and mouse brains were collected at CT22–01. Data are presented as mean ± SEM, with individual values shown (n = 4 per group). Statistical significance was determined by two-way ANOVA followed by Tukey’s post hoc analysis. *p < 0.05, **p < 0.01.

### SR8278 microinjection increases extracellular DA turnover without changing extracellular DA levels

To investigate whether REV-ERBα inhibition affects extracellular DA dynamics, we performed in vivo microdialysis in the striatum after acute SR8278 microinjection into the SN. Baseline samples were collected prior to drug administration, and dialysates were collected 1, 3, and 4 h after injection to measure extracellular DA, DOPAC, and HVA (Figure 5A). Extracellular DA levels did not differ comparable among groups, indicating that neither 6-OHDA lesion nor SR8728 treatment altered extracellular DA concentration (Figure 5B). In contrast, extracellular DOPAC and HVA levels were significantly reduced in 6-OHDA-lesioned mice compared with sham controls (Figure 5C and D). Notably, SR8278 treatment increased both metabolites in lesioned mice, restoring them toward control levels. The extracellular DA turnover rate, calculated as (DOPAC + HVA)/DA, was also reduced after 6-OHDA-lesion and increased following SR8278 treatment (Figure 5E). Together, these findings indicate that SR8278 does not alter extracellular DA levels but instead increases extracellular DA turnover, suggesting an improvement in DA metabolic dynamics following 6-OHDA lesioning.

**Figure 5.**
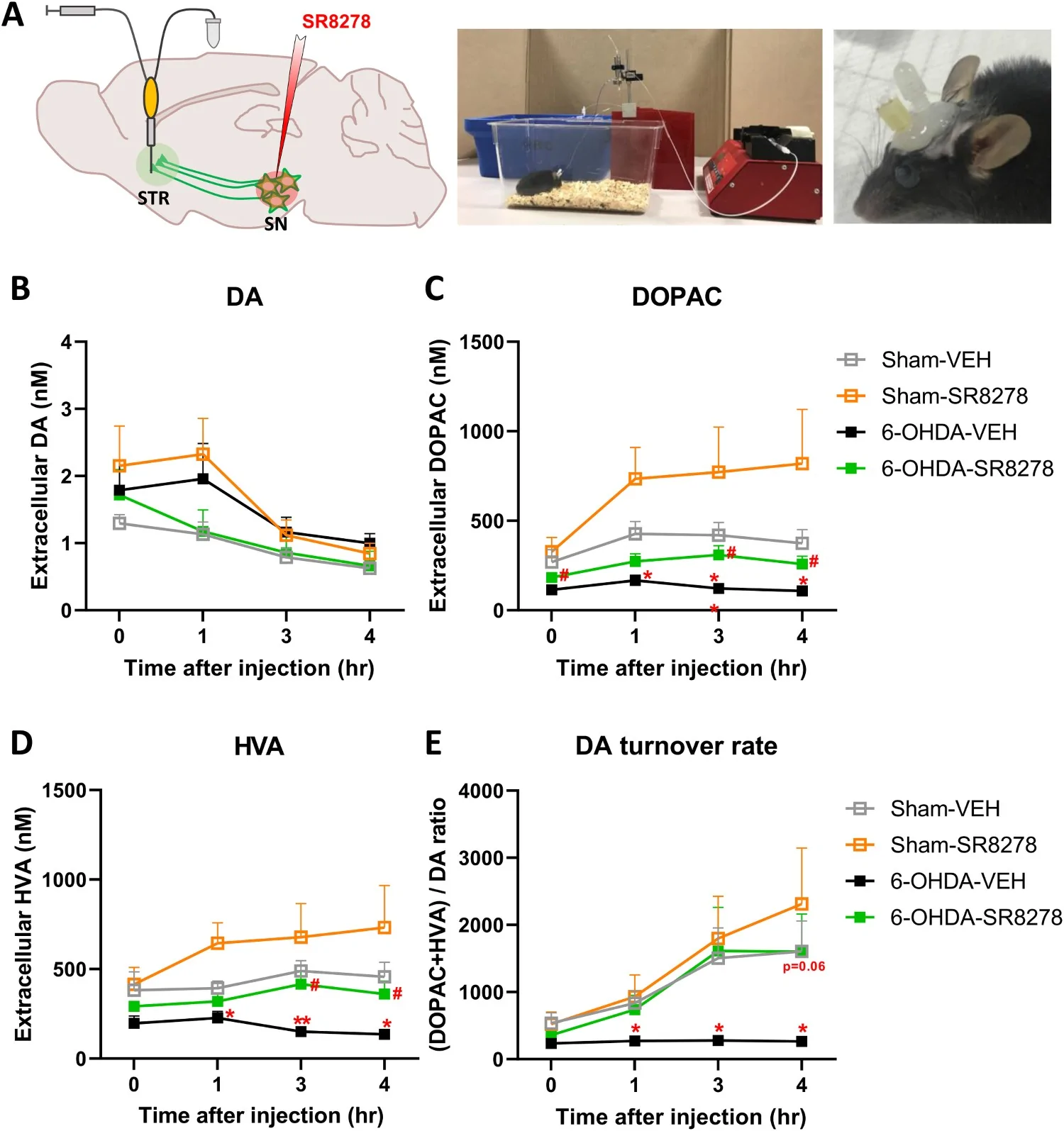
**SR8278 increases extracellular DA turnover in the striatum of 6-OHDA-lesioned mice.** (A) Experimental design for in vivo microdialysis. A microdialysis probe was placed in the striatum, and SR8278 was microinjected into the SN. Dialysates were collected before SR8278 infusion (baseline), and at 1, 3, and 4 h after treatment for liquid chromatography-tandem mass spectrometry (LC-MS/MS) analysis. (B-E) Time-course of extracellular DA, DOPAC, HVA, and DA turnover ratio ((DOPAC + HVA)/DA) in the striatum of sham- and 6-OHDA-lesioned mice treated with VEH or SR8278. SR8278 was administered at CT08 or CT20, and dialysates were collected during CT09–13 or CT21–01. Data are expressed as mean ± SEM, with individual time points shown. Statistics was determined by two-way repeated measure ANOVA followed by Fisher’s LSD test. Symbols indicate significant differences between groups (*p < 0.05, **p < 0.01 vs. sham-VEH; #*p* < 0.05 vs. 6-OHDA-VEH).

## Discussion

In this study, we demonstrate that acute pharmacological inhibition of REV-ERBα activity by SR8278 microinjection into the SN improved fine motor function in a 6-OHDA-lesioned mouse model of PD. This effect was accompanied by increased extracellular DA metabolites (DOPAC and HVA), indicating higher extracellular DA turnover in the striatum, while basal extracellular DA levels remained unchanged. SR8278 did not restore DA synthesis-related markers or tissue DA content in either the SN or the striatum.

SR8278 microinjection improved asymmetric forelimb use in the cylinder test in the 6-OHDA-lesioned mice and improved performance in the pole test in the MPTP model. In contrast, SR8278 did not affect rotarod performance. These behavioral assays are widely used to assess motor deficits in rodent PD models (Sheta et al. 2024). The cylinder test is sensitive to fine motor deficits after unilateral DAergic lesions (Schallert et al. 2000, Glajch et al. 2012), while the pole test reflects bradykinesia associated with nigrostriatal DAergic dysfunction (Matsuura et al. 1997). In contrast, the rotarod test depends on whole-body balance and coordination and may detect motor deficits that are not solely dependent on nigrostriatal DAergic function (Magen and Chesselet 2010). Together, these findings indicate that REV-ERBα antagonism improves fine motor function but does not alter motor coordination.

To explore the neurochemical basis of these behavioral effects, our analyses showed distinct changes in DA regulation at the tissue and extracellular levels in the 6-OHDA model. HPLC-ECD demonstrated a reduction in striatal DA content, whereas DA levels in the SN were relatively less affected. This pattern is consistent with previous research showing that DAergic terminals degenerate before nigral cell bodies (Sauer and Oertel 1994). Despite the loss of striatal tissue DA, in vivo microdialysis showed that basal extracellular DA levels remained unchanged, while extracellular DOPAC and HVA were significantly reduced in the striatum. Similar findings have been reported previously, where basal extracellular DA is maintained despite substantial loss of striatal tissue DA, while extracellular DA turnover is reduced (Robinson and Whishaw 1988, Bergstrom and Garris 2003). Although SR8278 did not alter tissue DA or tissue DA metabolite levels, it increased extracellular DOPAC and HVA without affecting extracellular DA levels in the striatum. These findings suggest that SR8278 is associated with a rebalancing of DA availability and clearance, potentially through changes in DA release, reuptake, and extracellular degradation. The increase in extracellular DA turnover was accompanied by improved fine motor function, suggesting that extracellular DA turnover may contribute to motor performance in the lesioned state. Because extracellular DA metabolism involves COMT- and MAO-dependent pathways in both neuronal and non-neuronal cells (Meiser et al. 2013), REV-ERBα antagonism may influence DA regulation through multiple cellular mechanisms. Further studies are needed to define the pathways through which REV-ERBα regulates extracellular DA dynamics in PD.

REV-ERBα is a pleiotropic nuclear receptor that acts across multiple cell types and has been linked to several pathological processes, including DAergic neuronal vulnerability and neuroinflammation (Kim, et al. 2018, Kim, et al. 2022, Kou, et al. 2022, Gupta, et al. 2024, Wang, et al. 2024). Previous studies have also reported that REV-ERBα regulates DAergic gene expression under physiological conditions (Chung, et al. 2014, Jager, et al. 2014). Our findings suggest that under parkinsonian conditions, REV-ERBα activity in the SN is more closely associated with extracellular DA turnover than with DA synthesis-related pathways. Indeed SR8278 has been reported to increase TH transcription in the 6-OHDA-lesioned VTA (Kim, et al. 2022). We did not observe a similar effect in SN. One possible explanation is that extensive neuronal loss in the lesioned SN limits REV-ERBα-dependent transcriptional response. In contrast, REV-ERBα antagonism appears to affect extracellular DA turnover without restoring DA synthesis or tissue DA content.

Our study demonstrates that REV-ERBα antagonism with SR8278 increases extracellular DA turnover and improves fine motor function in mouse models of PD. REV-ERBα is a core component of circadian clock and regulates a wide range of physiological and behavioral processes, including sleep (Amador et al. 2016, Mang et al. 2016) and mood regulation (Chung, et al. 2014, Kim, et al. 2022, Jang et al. 2023, Park et al. 2024). Together with our previous findings on mood-related behaviors, the current results suggest that REV-ERBα modulation may serve as a promising therapeutic target for both motor and circadian-related non-motor symptoms of PD.

## Supplementary Material

Supplementary Figure_revised ver.pdf
