# Supplementary material for: Pharmacological inhibition of REV-ERBα ameliorates motor deficits in the 6-OHDA-lesioned mouse model of Parkinson’s disease
Source: Anim Cells Syst (Seoul). 2026 Oct 15;30(1):558–71. doi: 10.1080/19768354.2026.2690728 (PMC13674654; doi:10.1080/19768354.2026.2690728)
Supplement: Supplementary file 1 [file TACS_A_2690728_SM4886.pdf]

Supplementary figure 1 related figure 1

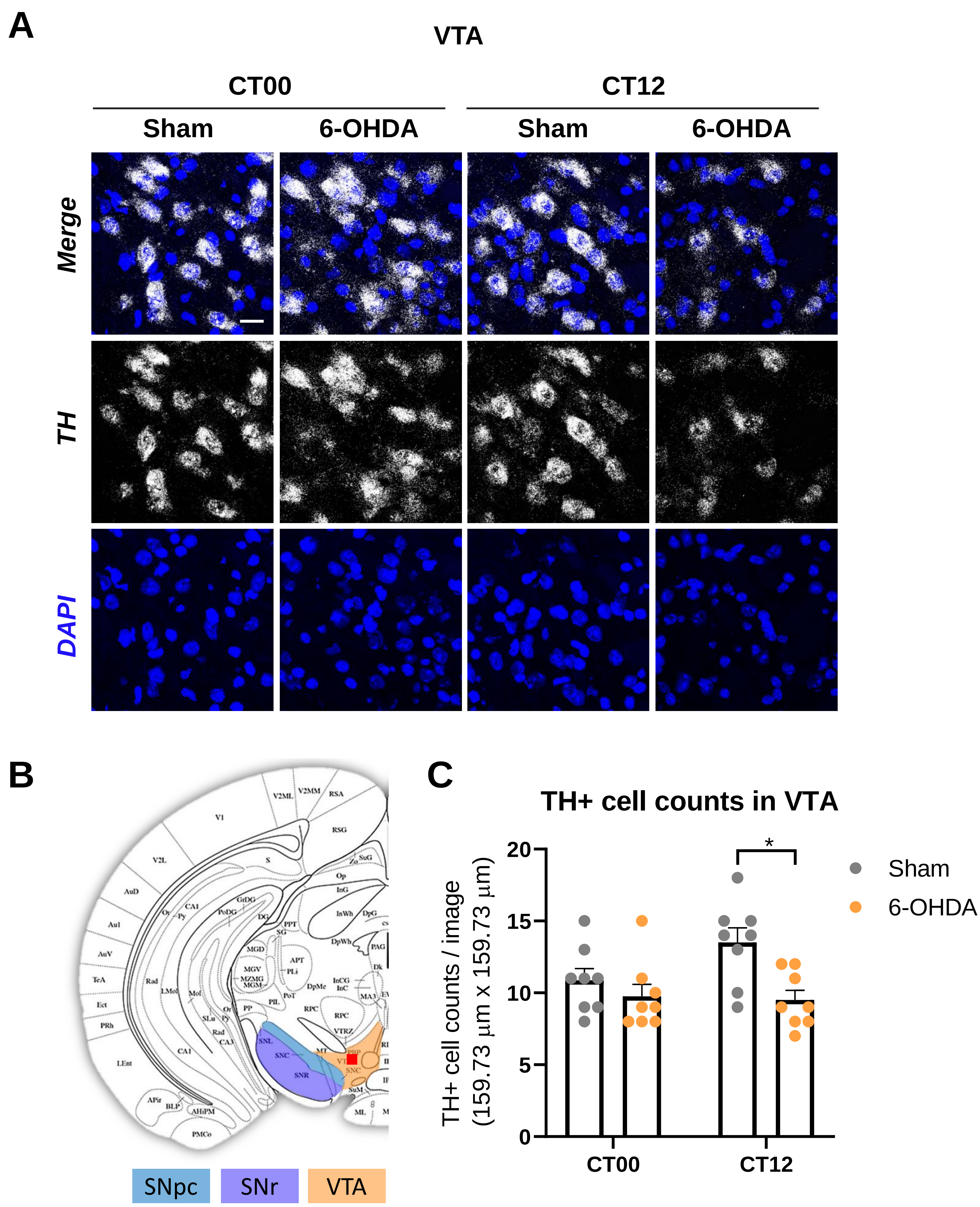

Supplementary Figure 1. VTA analysis following 6-OHDA lesion.
(A) Representative low-magnification FISH images of the VTA from sham and 6-OHDA-treated mice at CT00 and CT12. Merged images (TH mRNA, white; DAPI, blue), TH single-channel images, and DAPI single-channel images are shown. Scale bar, 20 μm. (B) A axinos and Franklin mouse brain atlas-based anatomical schematic indicating the imaging and quantification region within the VTA. Imaging and quantification were performed within the VTA in coronal sections corresponding to bregma −3.16 mm (AP), 0.5 mm (ML), 4.4 mm (DV), based on the Allen Brain Atlas. The imaging and quantification region is indicated in red. SNpc, substantia nigra pars compacta; SNr, substantia nigra pars reticulata; VTA, ventral tegmental area. (C) Quantification of TH-positive cell counts in the VTA from low-magnification images (159.73 μm × 159.73 μm field of view) Data are presented as mean ± SEM, with individual values shown. N=8. Statistical significance was determined by two-way ANOVA followed by Tukey's post hoc analysis. *$p<0.05$.

Supplementary figure 2 related figure 2

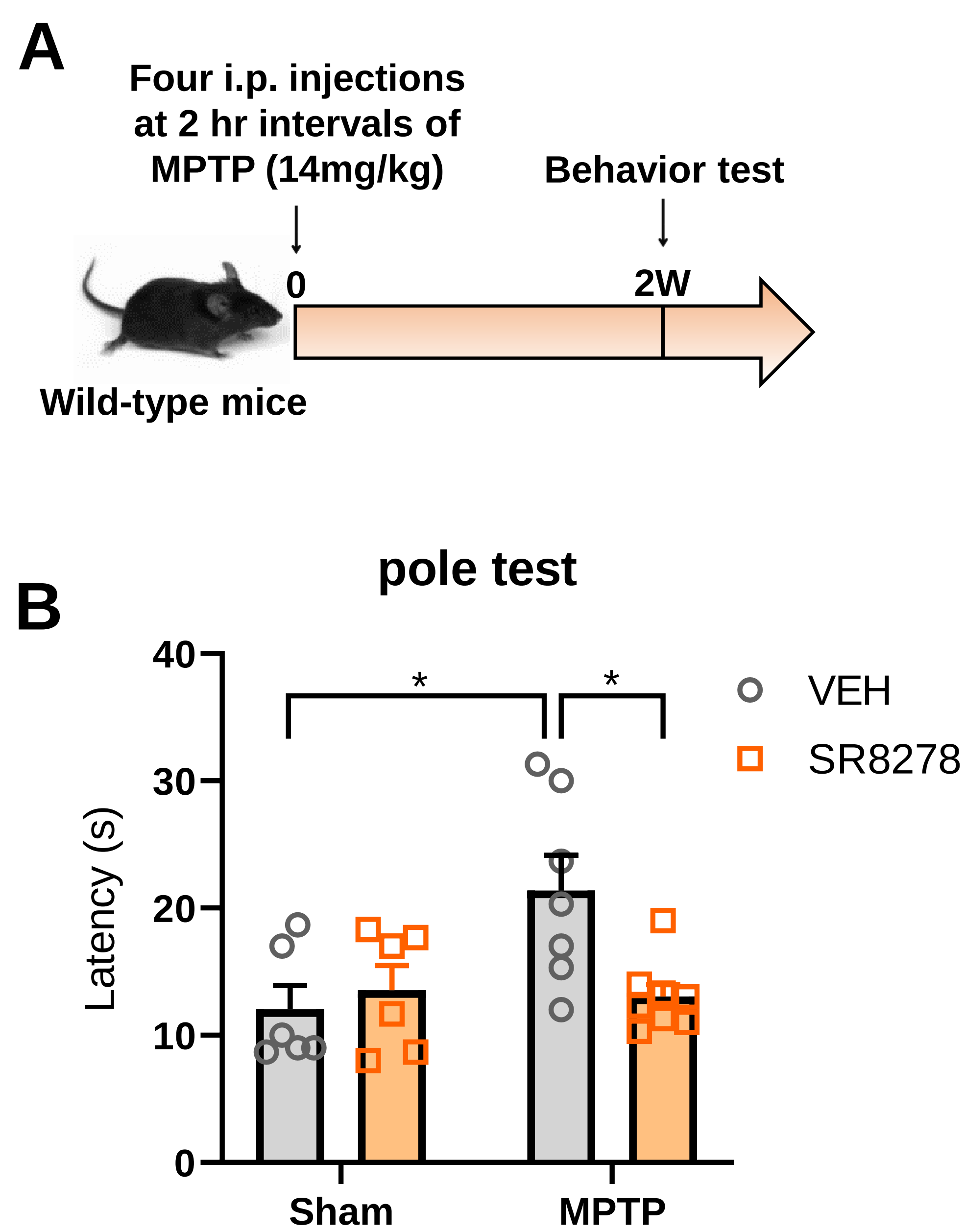

Supplementary Figure 2. SR8278 microinjection improves motor performance in the MPTP-induced PD model.

(A) Experimental timeline for behavioral assessment in mice subjected to MPTP lesioning. MPTP was administered at a dose of 14 mg/kg by four intraperitoneal (i.p.) injections at 2 h intervals over a single day. Following 2 weeks after injection, behavior test was performed 3 hr after SR8278 microinjection into the midbrain including SN. (B) Motor performance was evaluated using the pole test. Data are presented as mean ± SEM, with individual values shown. N = 6-8. Statistical significance was determined by two-way ANOVA followed by Tukey's post hoc analysis. *$p<0.05$.

Supplementary figure 3 related to figure 3

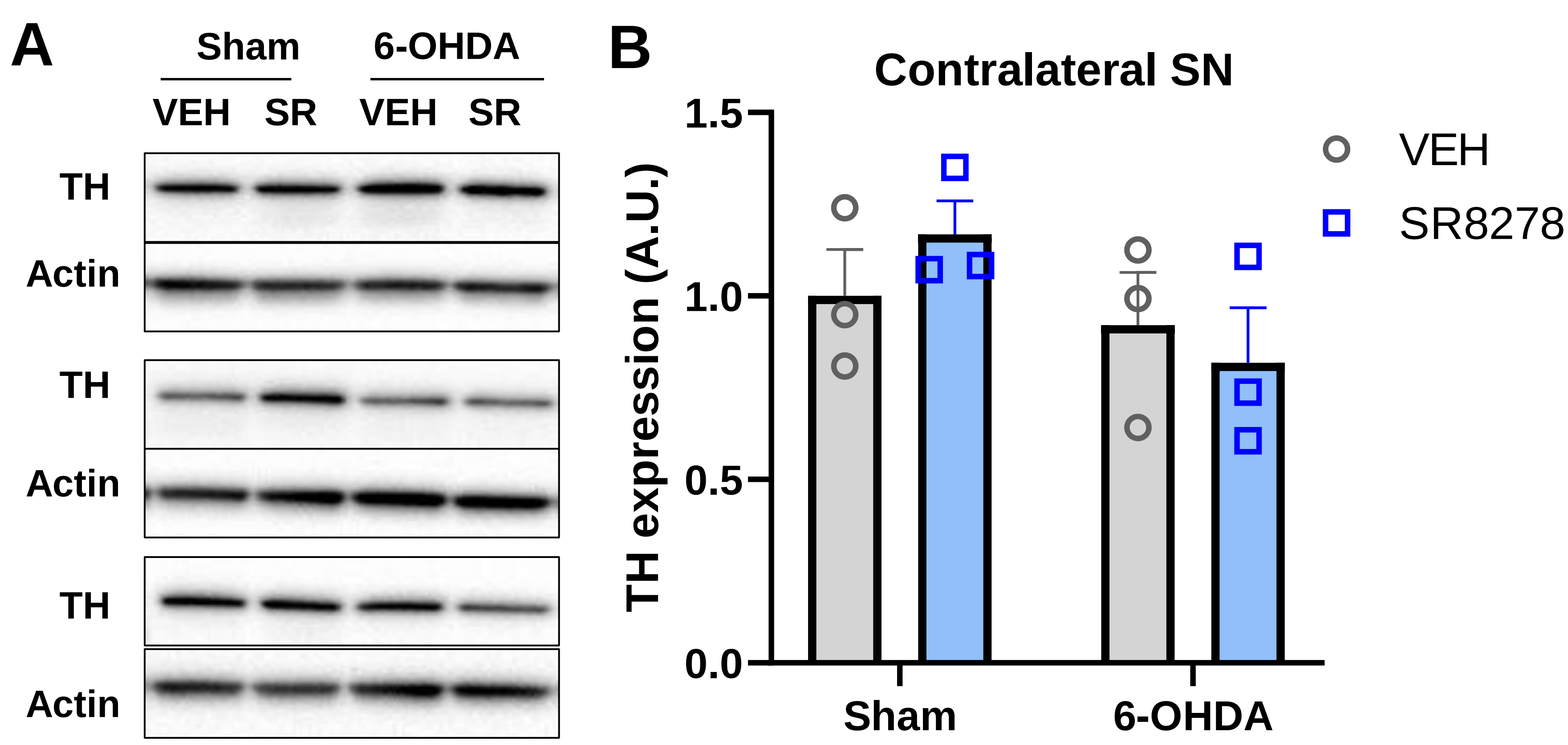

Supplementary figure 3. TH protein expression in the contralateral SN of 6-OHDA-lesioned mice.

(A) Representative western blot images showing TH from the contralateral SN of sham-VEH, sham-SR8278, 6-OHDA-VEH, and 6-OHDA-SR8278 groups. β-actin was used as loading control. (B) Densitometric quantification of TH normalized to β-actin. Data are presented as mean ± SEM, with individual values shown. N=3.

Supplementary figure 4 related to figure 4

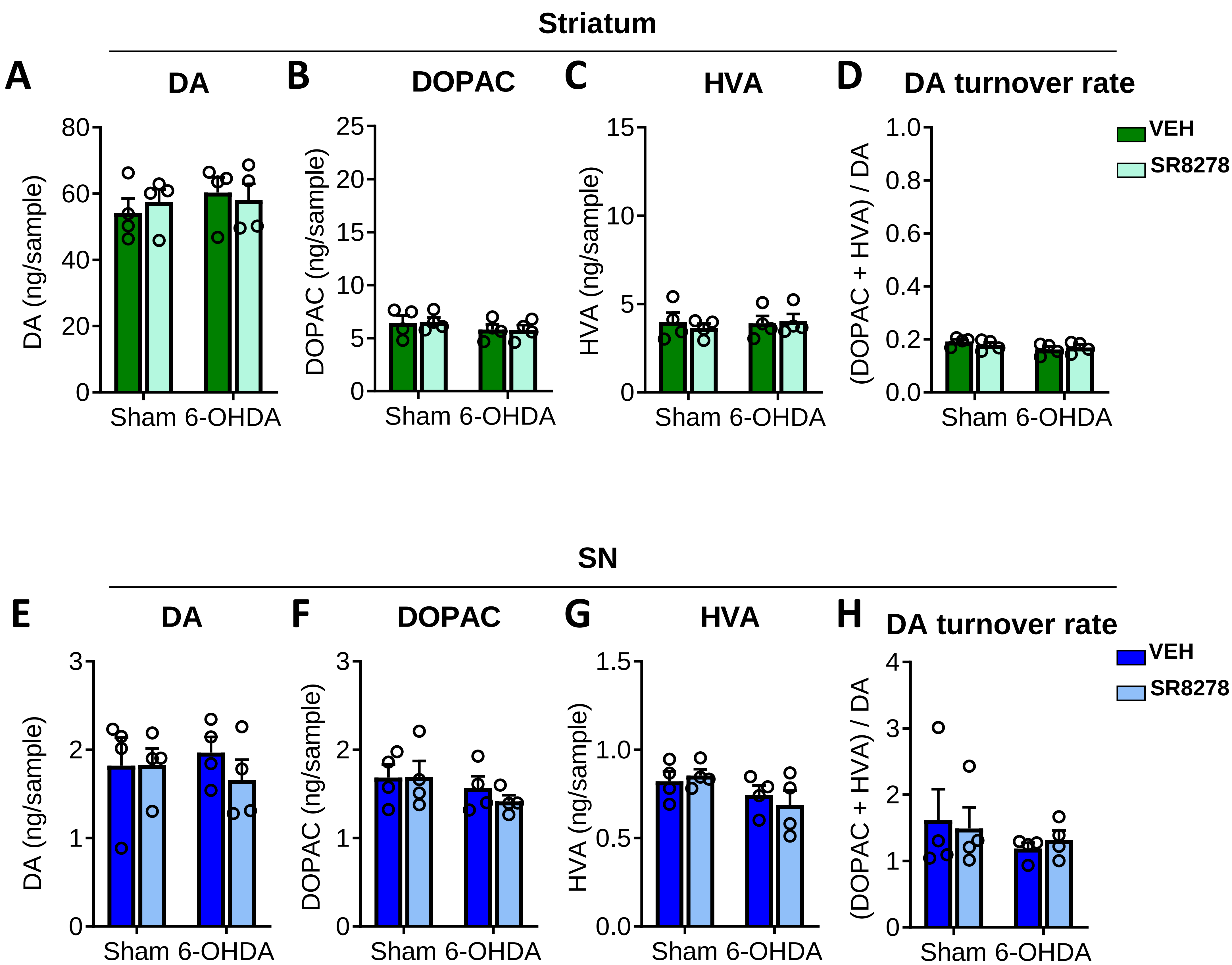

Supplementary figure 4. DA and its metabolite levels in the contralateral hemisphere of 6-OHDA-lesioned mice.

(A-D) Tissue DA, DOPAC, HVA, and DA turnover ratio ((DOPAC+HVA)/DA) in the contralateral striatum. Neurochemical analysis was performed using HPLC-ECD. Data are expressed as mean ± SEM, with individual values shown. N = 4. (E-H) Corresponding measurements in the contralateral SN. Data are expressed as mean ± SEM, with individual values shown. N = 4.
